# Social media to enhance engagement and science dissemination during in-person and virtual medical conferences: the SCMR 2020 and 2021 experiences: a report of the SCMR social media task force

**DOI:** 10.1186/s12968-021-00837-x

**Published:** 2022-03-07

**Authors:** Mrinali Shetty, Niti R. Aggarwal, Purvi Parwani, Chiara Bucciarelli-Ducci, Juan Lopez-Mattei, Andrew Choi, Lars Grosse-Wortmann

**Affiliations:** 1 University of Chicago (NorthShore) Program, Evanston, IL USA; 2 Department of Cardiovascular Disease, Mayo Clinic, Rochester, MN USA; 3 Division of Cardiology, Department of Medicine, Loma Linda University Health, Loma Linda, CA USA; 4 Royal Brompton and Harefield Clinical Partnership, Guys and St Thomas NHS Trust and King’s College, London, SW3 6NP UK; 5 Departments of Cardiology and Thoracic Imaging, UT MD Anderson Cancer Center, Houston, TX USA; 6 Division of Cardiology and Department of Radiology, The George Washington University School of Medicine, Washington, DC USA; 7 Division of Cardiology, Department of Pediatrics, Oregon Health and Science University, Portland, OR USA; 8 Department of Pediatrics, The Hospital for Sick Children, University of Toronto, Toronto, ON Canada

**Keywords:** Education, Conference, Social media, Twitter, Cardiac MRI, Influencers, SCMR

## Abstract

Most cardiac imaging conferences have adopted social media as a means of disseminating conference highlights to a global audience well beyond the confines of the conference location. A deliberate and thoughtful social media campaign has the potential to increase the reach of the conference and allow for augmented engagement. The coronavirus disease 2019 (COVID-19) pandemic triggered a radical transformation in not just the delivery of healthcare but also the dissemination of science within the medical community. In the past, in-person medical conferences were an integral annual tradition for most medical professionals to stay up to date with the latest in the field. Social distancing requirements of the COVID-19 pandemic resulted in either cancelling medical conferences or shifting to a virtual format. Following suit, for the first time in its history, the 2021 Society for Cardiovascular Magnetic Resonance (SCMR) annual meeting was an all-virtual event. This called for a modified social media strategy which aimed to re-create the sociability of an in-person conference whilst also promoting global dissemination of the science being presented. This paper describes the employment of social media as well as the evolution through the SCMR scientific sessions for 2020 and 2021 that serves as a model for future cardiovascular conferences.

## Introduction

Social media (SoMe) platforms have seen widespread adoption by the medical community and have changed the way many professional societies disseminate new science and information [1, 2]. The use of Twitter during medical conferences has gained significant traction secondary to the platform’s ability to reach a wide audience without geographic barriers, in real time and at the user’s convenience [1, 3–5]. The scientific content is distilled into a ‘tweet’ (a microblog of 280 characters) or a thread (a series of tweets), which is then broadcast globally. Such SoMe campaigns have been shown to amplify the educational and research endeavors of conferences, and allow real-time participation of users [6]. The Society for Cardiovascular Magnetic Resonance (SCMR) is the largest association of experts in cardiovascular magnetic resonance (CMR). For the SCMR Scientific Sessions 2020 an effective social media strategy to engage CMR users worldwide was established. On the heels of SCMR 2020, the emergence of coronovirus disease 2019 (COVID-19) pandemic heralded a paradigm shift in the delivery and dissemination of medical knowledge. The tradition of physicians gathering in convention centers or hotels to educate, be educated and to network was paused. Apart from the scholarship, the appeal of such colloquiums was in the esprit de corps from which are born new academic collaborations, mentorships, research, and employment opportunities. In response to the pandemic, medical conferences that were not cancelled shifted to a virtual format. SCMR has placed a special focus on furthering the social media interactions and engagement to enhance the educational opportunities for CMR users worldwide.

The aim of this paper is to describe how a dedicated SoMe strategy enhances the experience at both in person and virtual conferences, using the examples of SCMR 2020 and 2021 annual scientific sessions.

## The role of social media in medical knowledge dissemination

### The #SCMR2020 and #SCMR2021 SoMe experience

Prior to the SCMR annual sessions 2020 and 2021, a social media task force comprised of the program chair, chief executive officer (CEO) as well as invited SCMR committee and staff members was created. The official conference hashtag (#SCMR2020 and #SCMR2021 for the respective years) was prospectively registered with a commercial platform for healthcare SoMe analytics (Symplur Signals, Pasadena, California, USA). The inaugural SoMe campaign included the following pre-planned activities which were highlighted on SCMR’s official Twitter account (*@SCMRorg*), emailed to members via email lists, and highlighted during the Program Chair’s remarks in the opening plenary.

The following strategies were employed to maximize the impact of the SoMe campaigns:

#### Increasing speaker engagement

Leading up to the meeting, thought leaders within the CMR community, including all speakers and moderators of the annual session were encouraged to join Twitter to promote virtual engagement with attendees. Conference faculty were asked to list their Twitter handle on their slides and on the conference app. One week prior to the start of the meeting, selected presentations and their speakers were introduced and highlighted on Twitter. For these talks, summary points created by the presenters were tweeted immediately following the completion of the presentations.

#### Topic highlight videos

Prior to SCMR 2021, SoMe ambassadors interviewed leaders in the field about high yield topics such as COVID myocarditis, artificial intelligence in CMR. The video clips of these interviews were posted on the Society’s YouTube channel “SCMR HQ” and links were widely shared on various social media platforms including Twitter, Facebook, WhatsApp and LinkedIn [7].

#### Social media ambassadors

**Fig. 1:**
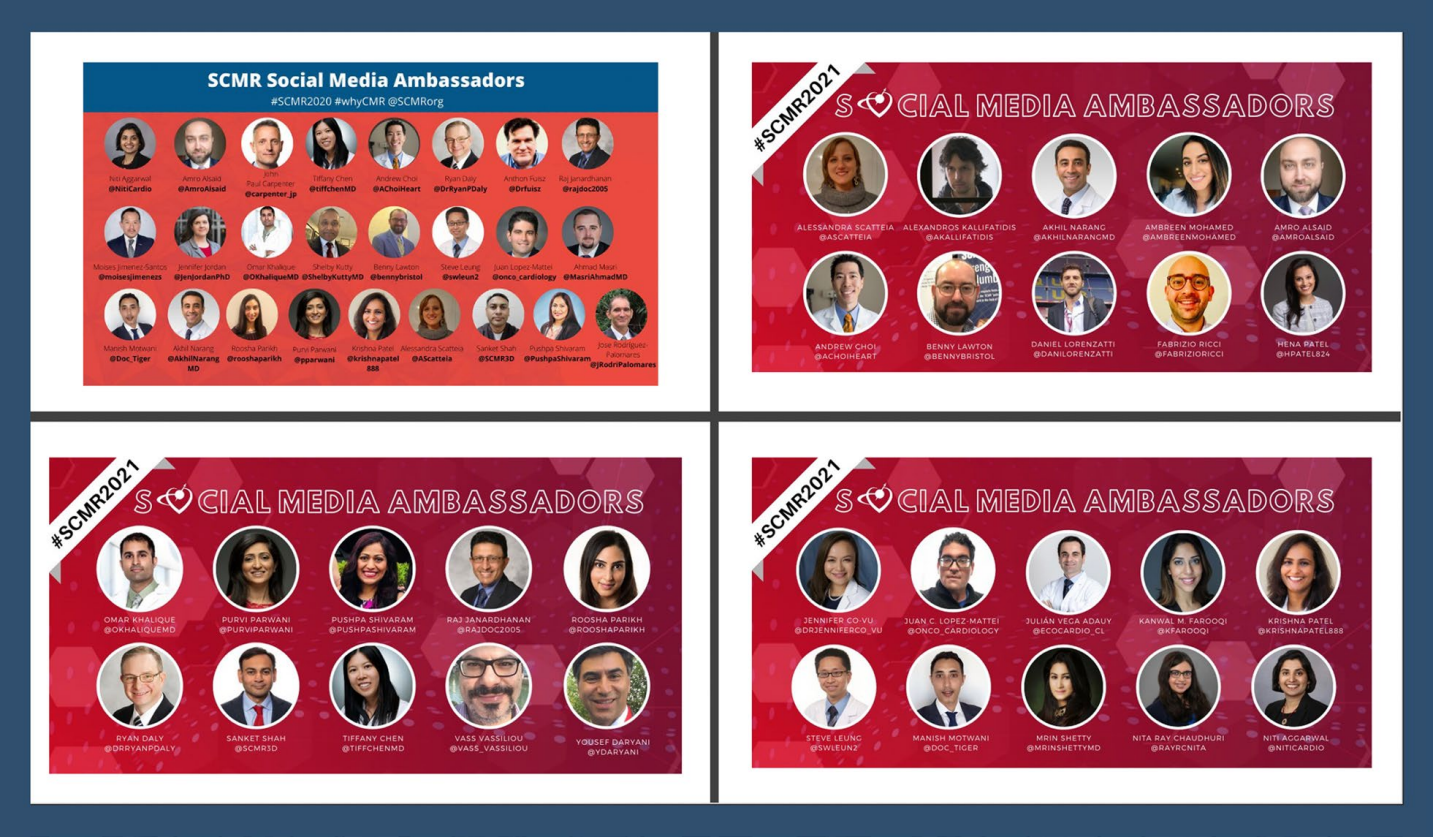
#SCMR2020 and #SCMR2021 SoMe Ambassadors: a team of cardiovascular magnetic resonance (CMR) experts with an established Twitter presence from the meeting registrants were invited to serve as social media ambassadors

CMR experts with an established SoMe presence and attending the meeting in person were invited to serve as ‘SoMe Ambassadors. Twenty-five ambassadors in 2020 and 30 ambassadors in 2021 were selected (Fig. 1) and assigned to cover 3–5 sessions each. Each SoMe Ambassador drew attention to the assigned session by announcing it on Twitter and summarizing the key take-home messages for the session. For SoMe Ambassadors, participation in the meeting was a rewarding opportunity to gain visibility in the academic community, network and to learn from the experts.

#### Twitter clinic

Recognizing the wide variation in Twitter proficiency amongst SCMR attendees, during SCMR 2020 live conference, a Twitter Clinic was set-up where attendees could stop by during conference breaks for customized one-on-one learning opportunities to get started on Twitter or troubleshoot technical challenges. To emulate the SCMR 2020 Twitter clinics virtually, during SCMR 2021, brief videos were created by two #SCMR2021 SoMe Ambassadors to introduce Twitter to attendees who were not familiar with the platform. The videos were advertised in the SCMR newsletter prior to the SCMR 2021 conference [8].

#### Social media session

During SCMR 2020, a dedicated 45-min session encompassing SoMe usage, the basics, advantages and challenges was hosted and included the following presentations:

1. Social Media 101
2. Advancing My Imaging Career: Social Media No Longer Optional?
3. How Can We Push CMR Through Social Media?

#### Tweet-up

At SCMR 2020, a social gathering or “Tweet-Up” was organized to facilitate in-person interactions and collaborations between Twitter participants and Society leaders. This venue also served as an awards ceremony for recognition of the SoMe Ambassadors, and for the winners of the Ribbon contest (see below).

During SCMR 2021, a virtual version of the tweet up was hosted on an application called Gather Town, a video chat platform where users interact through avatars in virtual rooms [9]. Both of these events were well-attended by SoMe participants, particularly by those in training and in early career.

#### ‘Create your own ribbon’ contest

**Fig. 2:**
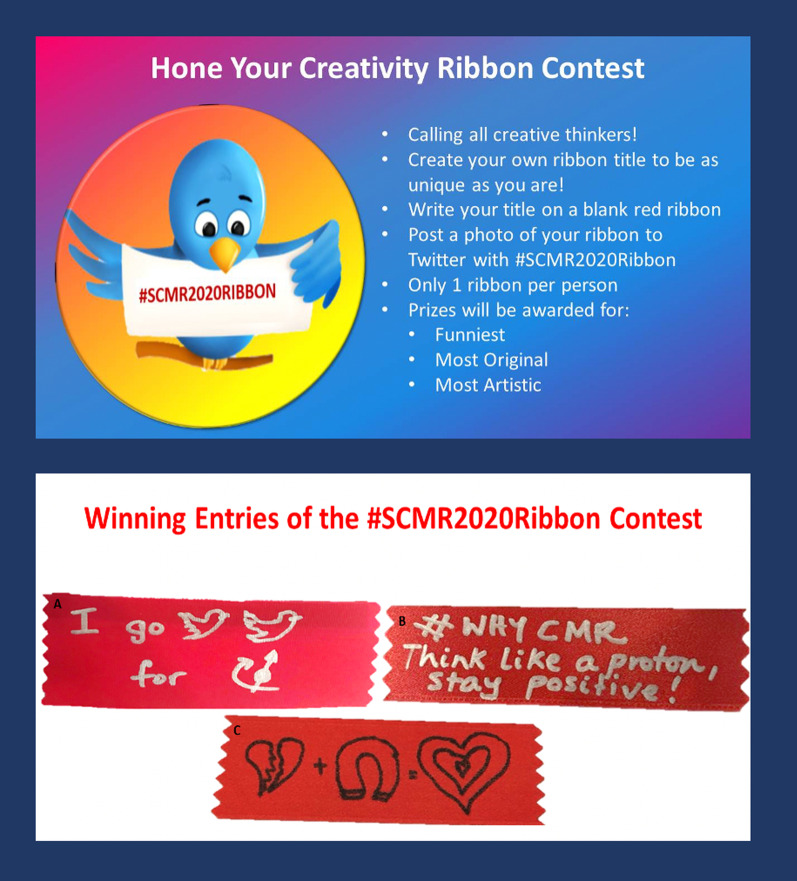
Top panel: Society for Sardiovascular Magnetic Resonance (SCMR) 2020 Ribbon Contest announcement and rules. Participants were encouraged to create their own ribbon and post on Twitter using a designated hashtag. Bottom panel: award winning entries of the #SCMR2020Ribbon contest. Prizes were awarded for the **a** the funniest **b** most original and **c** most artistic tweets

To foster delegate engagement and to encourage Twitter usage, during SCMR 2020, a congenial ‘create your own ribbon’ contest was announced during the opening plenary. Signs posted at the ribbon station encouraged participants to create an innovative, fun and/or unique ribbon, and post it on Twitter, labelling it with the hashtag #SCMR2020Ribbon. Prizes were awarded for the funniest, most original and most artistic entries during the tweet-up (Fig. 2).

#### Fitness initiatives on the virtual platform

**Fig. 3:**
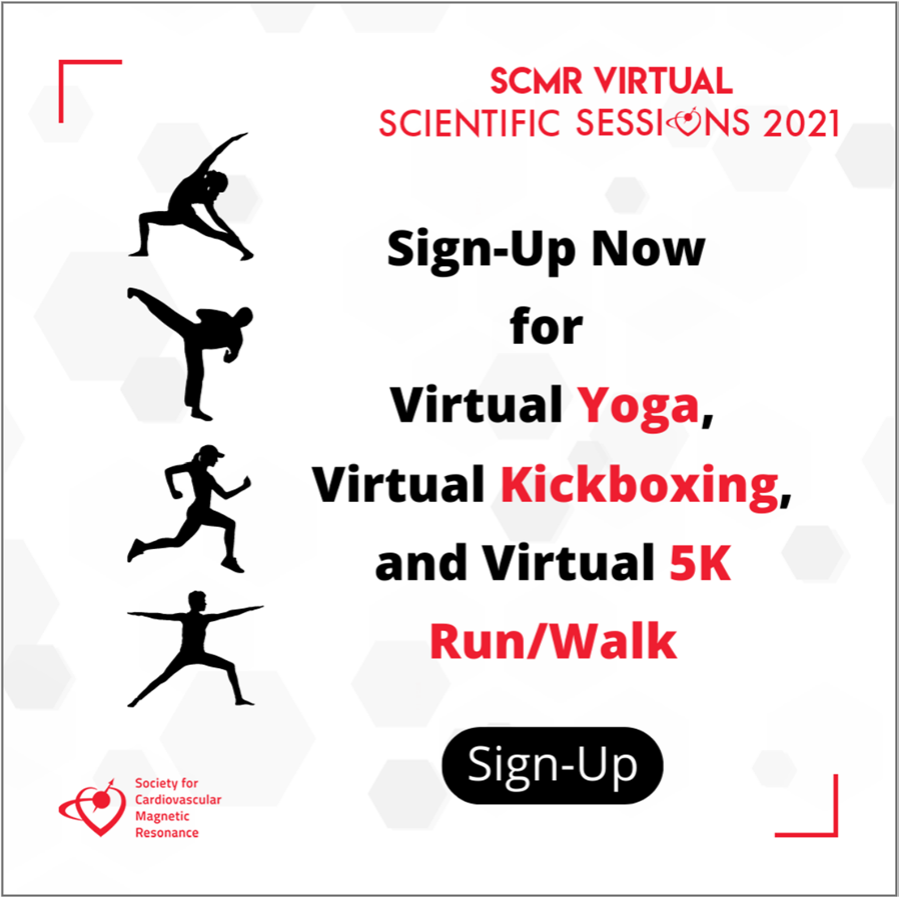
The fitness initiatives at SCMR 2021

Given the lack of in-person interaction at SCMR 2021, SoMe friendly efforts to increase attendee engagement such as the fitness initiative which encompassed several different sessions was organized. Virtual yoga and Muay Thai kickboxing classes were conducted over Zoom. Coordinated classes on the Peloton bike were another offering (Fig. 3). On the Peloton app, a CMR community members could search one another and do group rides using the hashtag #SCMR.

#### Promoting inclusivity: #CMRTeamSpirit

**Fig. 4:**
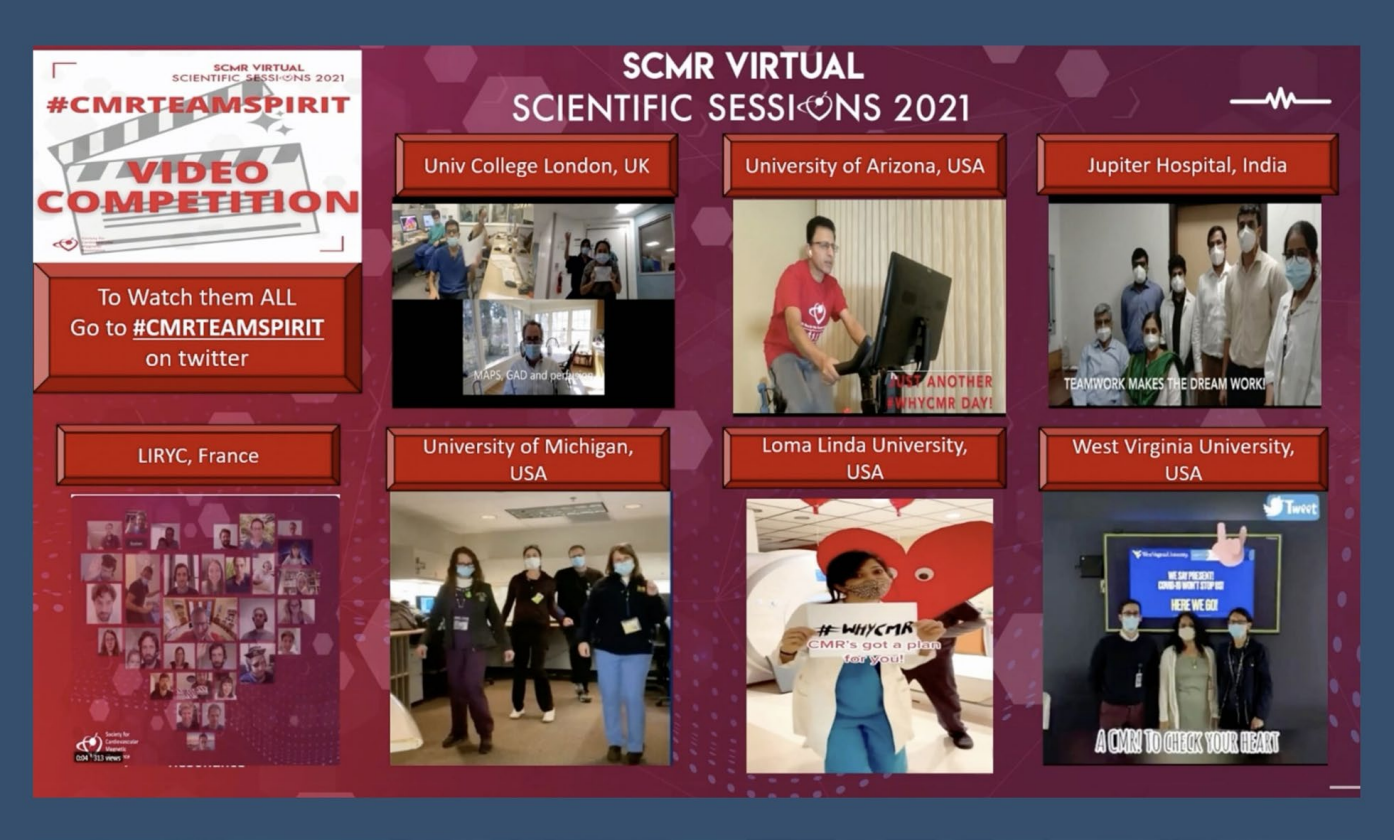
Some of the video submissions for the #CMRTeamSpirit competition, aimed at showcasing different CMR labs around the world and appreciating the work of their various team-members

Recognizing that the success of a CMR service lies in team effort from physicians to nurses to technologists, SCMR 2021 hosted the virtual #CMRTeamSpirit competition to give teams across the world a chance to come together and showcase their creative skills while having a good time. The competition invited participants to submit a minute-long video highlighting their CMR lab’s team spirit while maintaining social distancing and COVID precautions. The top three videos were then played on the final day of the conference before a winner was announced. These videos were also uploaded on Twitter and received with an enthusiastic response from the imaging community (Fig. 4).

#### Emphasis on social media to augment industry engagement

**Fig. 5:**
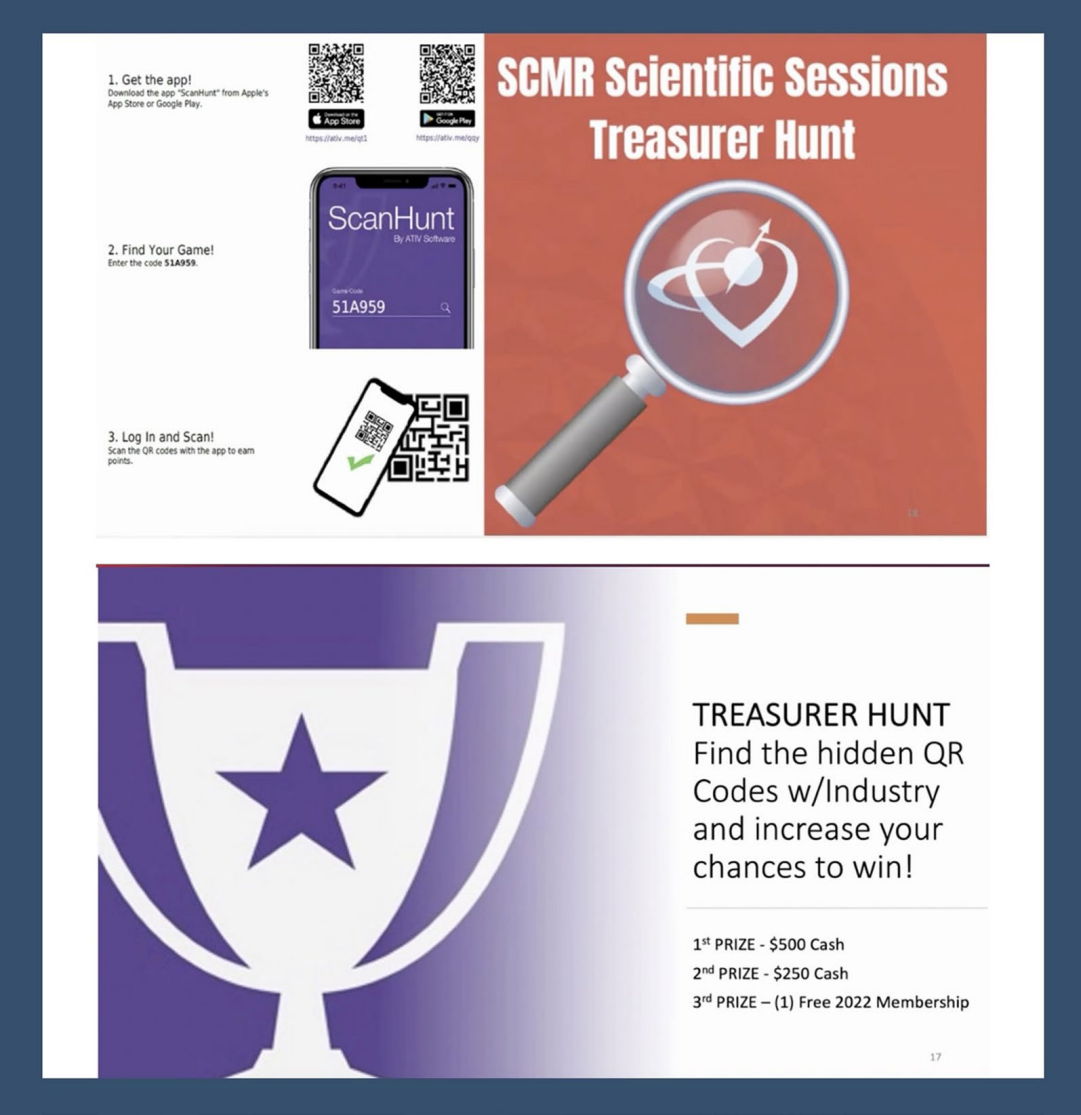
Promoting engagement with industry through the virtual “treasure-hunt”

Industry interaction has been limited in the virtual conference format. To overcome this, during SCMR 2021, the social media taskforce established a virtual treasure hunt using the “ScanHunt” app to gamify connectivity and updates with industry representatives. The top scorers won cash prizes and free SCMR membership (Fig. 5).

## Meeting engagement on twitter

1. *SCMR 2020*

Twitter activity was analyzed from the time of registration of the #SCMR2020 hashtag to 6 weeks from completion of the conference (1/1/2020-4/1/20) using the Symplur analytics platform to capture the extent and impact of SoMe activities using criteria that has been previously published [10].

**Fig. 6:**
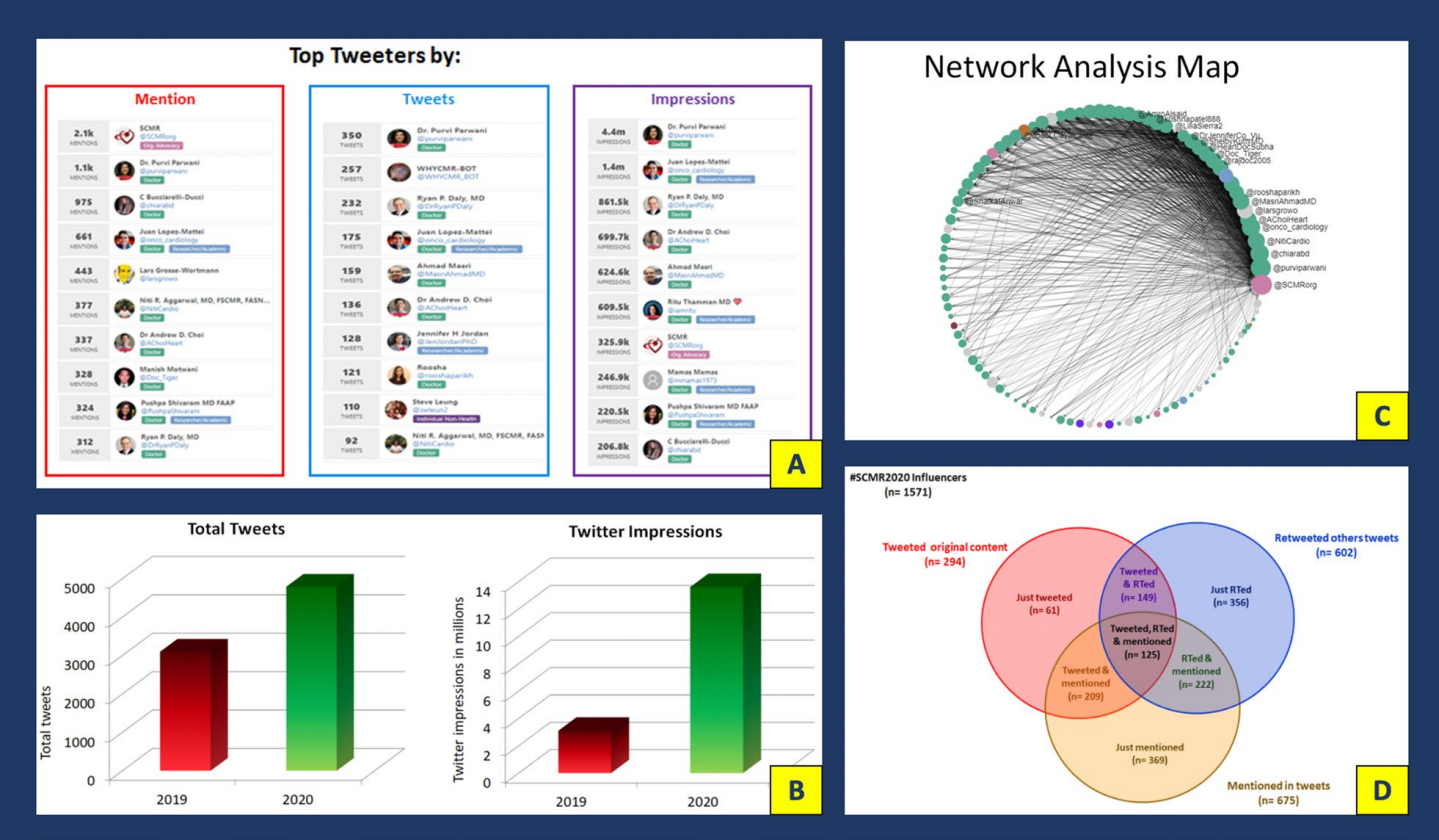
**A** The top 10 influencers of #SCMR2020 listed by mention, tweets and impressions. **B** SCMR2020 Twitter Activity compared to SCMR 2019—during SCMR 2020, there were 4792 tweets representing a 55% increase in the total number of tweets compared to the SCMR 2019 annual meeting. This resulted in a fourfold increase in the number of impressions, to 13.6 million. **C** Network analysis map of the #SCMR2020 data summarizing the activity of the top 100 participants, demonstrating the interaction between participants with connecting lines, and highlighting the participants most influential to the conversations. The impact of the influencers to the conversation is reflected by the size of the node. Organizations are listed in *pink*, and individuals in *green*. **D** a Venn diagram of the #SCMR2020 influencers—the total number of, and interaction between the tweeters, retweeters and mentioned accounts. RTed = retweeted

Over the 3-month capture period, a total of 747 active Twitter users generated 4792 tweets (of which 70% were retweets) using the official #SCMR2020 hashtag, resulting in 13.6 million impressions. This represented a 55% increase in the total number of tweets compared to the 2019 meeting and a fourfold increase in the number of impressions (Fig. 6B). The growth in number of tweets occurred with only a modest increase in registration between SCMR 2019 and SCMR 2020. For SCMR 2020, there were 1571 active tweeters, which exceeded the total registered delegates. Users that generated an original tweet or retweeted represented 51.3% of the total registered delegates. The 1423 (69.7%) original tweets by 294 users accounted for 3.1 million (30.0%) of the all the impressions. In contrast, retweets (comprising 70.3% of the total tweets) from 600 users resulted in the remaining 70% of impressions. The tweets that could be localized based on the information in the user profile (64.3% of total) originated from 39 countries across five continents, indicating the international reach of the conference. The top tweeters by number of tweets, impressions, and mentions were recognized (Fig. 6A). A network analysis map, summarizing the activity of the top 100 tweeters, is depicted in Fig. 5C. Figure 6D demonstrates the breakdown of the tweeters by the type of influencer: tweeter, retweeter, or mentioned account. The largest category was those that were mentioned (675 accounts) followed closely by those that retweeted (602 accounts).

2. *SCMR 2021*

**Fig. 7:**
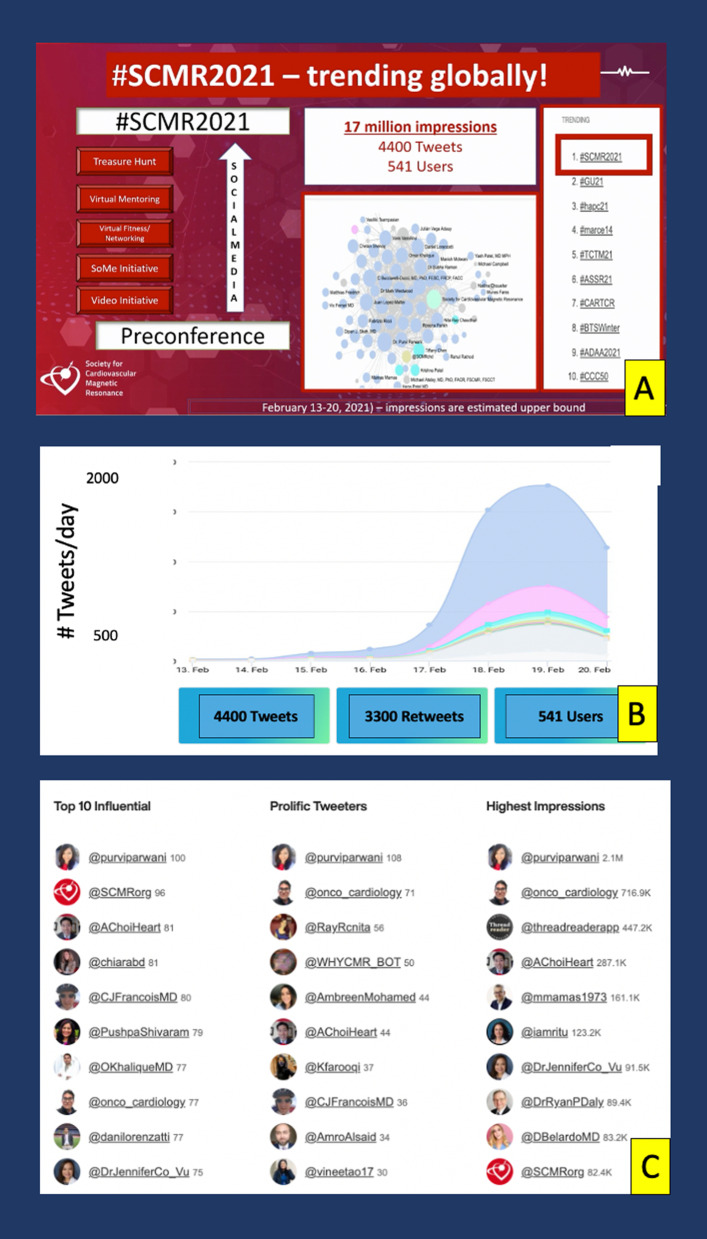
**A** The success of the social media campaign evidenced by #SCMR2021 becoming the highest trending medical conference hashtag of its time. **B** Rise in number of tweets by day in the month of February 2021. **C** Lists of top 10 most influential tweeters, most prolific tweeters and those who generated the highest impressions

Once again, Twitter activity was analyzed from the time of registration of the #SCMR2021 hashtag to six weeks from completion of the conference using the Symplur analytics. Over the 3-month capture period, 4400 tweets were generated from 541 users across every continent, with 17 million impressions created, making #SCMR2021 the highest trending medical conference of its time, and a truly global event (Fig. 7).

## Social media adoption in conferences

One of the highlights of digital technology during the 2020 SCMR conference was the emphasis on social media, in 2021 it was centered around hosting an all-virtual conference, a first for the Society. Both years, social media played a key role in boosting engagement. In fact, healthcare professionals and organizations are increasingly engaging on Twitter given that there is no user fee, it is easy to use, it allows real-time interactions and it has a global reach. This has resulted in the widespread adoption of SoMe by most contemporary cardiology conferences [3, 6, 11, 12. An organized SoMe campaign is thus well suited to expand the reach beyond the physical conference without substantially expanding the budget. In addition to dissemination of science and knowledge, the sharing of group photos and healthy discussions build camaraderie and the community.

## Conclusion

Social media has established itself as a powerful tool for the dissemination of knowledge, increase engagement at a scientific conference and for fostering a sense of community. It is thus an excellent auxiliary to the in-person and virtual medical conference experience and has been widely adopted by the cardiology community. SCMR integrated social media with its annual conferences starting in 2019. The COVID-19 pandemic heralded a transformation in the delivery and dissemination of science. Medical conferences that were not cancelled evolved to a virtual model and social media played an even more important role in enhancing engagement and promoting learning as demonstrated by the social media metrics of SCMR 2021, the Society’s first virtual-only conference. With the upcoming virtual-only 2022 SCMR Annual Scientific Sessions, it is likely that social media will play an increasingly important role in knowledge dissemination and attendee engagement.

**Fig. 8:**
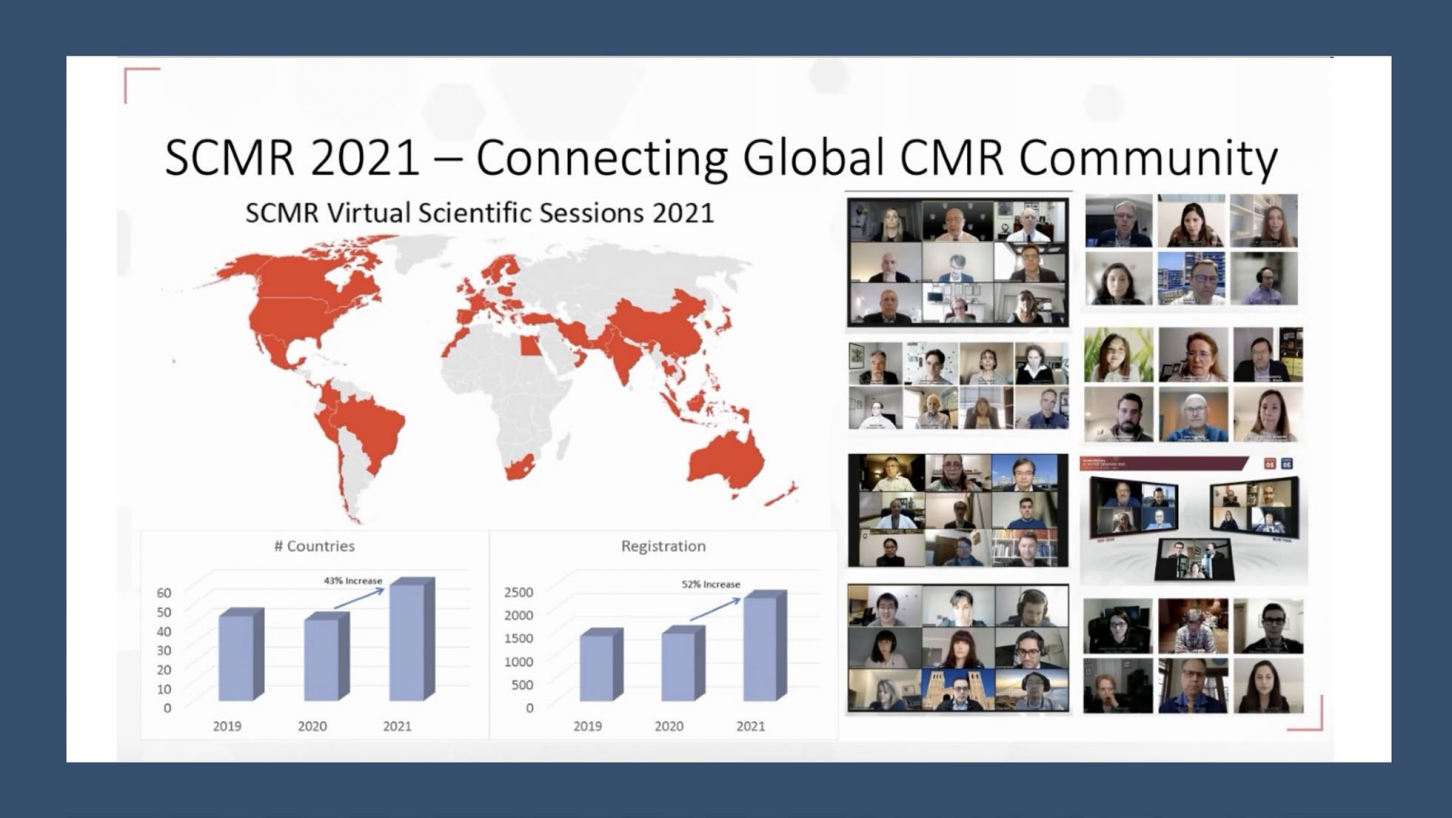
SCMR 2021 was successful in connecting the global CMR community with increased outreach than any prior year with a 52% increase in number of registrants and a 43% increase in number of participating countries

To demonstrate the exponential rise in popularity of SoMe over the past few years, during SCMR 2020, there were 4792 tweets representing a 55% increase in the total number of tweets compared to the 2019 meeting. This resulted in a fourfold increase in the number of impressions, to 13.6 million. SCMR 2021 generated 17 million Twitter impressions world-wide, with an increase of 3.9 million impressions from 2020 (Fig. 8). As this evolution continues, further research is warranted to better understand user preferences, engagement predilections and learning styles to enhance the attendee experience.

## Data Availability

Data sharing not applicable to this article as no datasets were generated or analyzed during the current study.
